# Experimental Models of Polycystic Kidney Disease: Applications and Therapeutic Testing

**DOI:** 10.34067/KID.0000000000000209

**Published:** 2023-07-07

**Authors:** Cynthia J. Sieben, Peter C. Harris

**Affiliations:** Division of Nephrology and Hypertension, Mayo Clinic, Rochester, Minnesota

**Keywords:** ADPKD, polycystic kidney disease

## Abstract

Polycystic kidney diseases (PKDs) are genetic disorders characterized by the formation and expansion of numerous fluid-filled renal cysts, damaging normal parenchyma and often leading to kidney failure. Although PKDs comprise a broad range of different diseases, with substantial genetic and phenotypic heterogeneity, an association with primary cilia represents a common theme. Great strides have been made in the identification of causative genes, furthering our understanding of the genetic complexity and disease mechanisms, but only one therapy so far has shown success in clinical trials and advanced to US Food and Drug Administration approval. A key step in understanding disease pathogenesis and testing potential therapeutics is developing orthologous experimental models that accurately recapitulate the human phenotype. This has been particularly important for PKDs because cellular models have been of limited value; however, the advent of organoid usage has expanded capabilities in this area but does not negate the need for whole-organism models where renal function can be assessed. Animal model generation is further complicated in the most common disease type, autosomal dominant PKD, by homozygous lethality and a very limited cystic phenotype in heterozygotes while for autosomal recessive PKD, mouse models have a delayed and modest kidney disease, in contrast to humans. However, for autosomal dominant PKD, the use of conditional/inducible and dosage models have resulted in some of the best disease models in nephrology. These have been used to help understand pathogenesis, to facilitate genetic interaction studies, and to perform preclinical testing. Whereas for autosomal recessive PKD, using alternative species and digenic models has partially overcome these deficiencies. Here, we review the experimental models that are currently available and most valuable for therapeutic testing in PKD, their applications, success in preclinical trials, advantages and limitations, and where further improvements are needed.

## Introduction

Polycystic kidney diseases (PKDs) can be broadly categorized into two main groups: nonsyndromic (affecting mainly the kidney and liver) and syndromic (affecting numerous organ systems). PKDs are considered ciliopathies because of defects in the functioning of primary cilia—data first provided by a study of *Caenorhabditis elegans*—with PKD proteins often localized to the cilium or basal body.^1,2^ The nonsyndromic PKD forms include autosomal dominant PKD (ADPKD) and autosomal recessive PKD (ARPKD). ADPKD is the most common (approximately 1:1000) and is typically adult-onset; *PKD1* (approximately 79%) and *PKD2* (approximately 15%) are the major genes, but mutations to at least six other loci are minor causes (*ALG5*, *ALG8*, *ALG9*, *GANAB*, *DNAJB11*, and *IFT140*).^3–11^ *PKD1* and *PKD2* encode polycystin-1 and -2 (PC1 and PC2), which form a functional complex at the primary cilium. Within ADPKD, there is considerable phenotypic heterogeneity ranging from limited cyst development and normal kidney function in old age to very early-onset disease, including neonatal lethality, with genic and allelic factors important.^12,13^ ADPKD is typically monoallelic, but biallelic inheritance has been rarely described, including in very early-onset presentations.^14–17^ Most patients with ADPKD become hypertensive; liver cysts are common and sometimes result in clinically significant polycystic liver disease (PLD); and there is an increased predisposition to intracranial aneurysms.^3,18^ ARPKD has an incidence of approximately 1:20,000, leads to neonatal lethality in approximately 25% of patients, and is mainly caused by biallelic mutations in *PKHD1*.^19–26^ ARPKD is typically characterized by massively enlarged cystic kidneys *in utero* or perinatally that can result in childhood ESKD and congenital hepatic fibrosis (CHF).^20–24,27–29^ There is also phenotypic heterogeneity in ARPKD, partly due to allelic effects, with some patients presenting as adults with more prominent CHF.^30^

Syndromic PKDs are rarer and usually recessively inherited, including Meckel syndrome, Joubert syndrome (JBTS), nephronophthisis (NPHP), and Bardet-Biedl syndrome (BBS).^31–35^ These diseases exhibit significant phenotypic and genetic heterogeneity and overlap; >10 different genes are mutated in each syndrome.^31,36^ The broad range of phenotypes likely reflects the role of primary cilia in orchestrating multiple signaling pathways, especially during development. However, each syndrome has distinct features. Meckel syndrome is the most severe with encephalocele or other central nervous system defects, CHF, and polydactyly and causes perinatal lethality.^31,32,37^ Whereas NPHP, JBTS, and BBS are typically childhood disorders associated with tubulointerstitial nephritis and cysts (NPHP), with the addition of cerebellar vermis hypoplasia, polydactyly, and CHF (JBTS), or cognitive impairment and obesity (BBS).^31,32^

Experimental models of these disorders in animals and from human patient material (*in vitro*) have arisen by spontaneous mutations (generated by nonspecific mutagenesis and screening) and generated by homologous recombination or more recently gene editing tools, such as Clustered Regularly Interspaced Short Palindromic Repeats (CRISPR)/Cas9 (*in vivo*). These models have been invaluable for understanding PKD pathogenesis, disease etiology, genetic interactions, and testing therapeutic candidates. Developing models that properly recapitulate organ properties (*in vitro*) and the human disease phenotypes (*in vivo*) has been a challenge, but numerous positive preclinical studies have been performed *in vitro* and in orthologous and nonorthologous animal models, even if the number progressing to clinical trials and US Food and Drug Administration approval has been more limited. Here, we review the orthologous *in vitro* and *in vivo* models suitable for therapeutic testing that are currently available for nonsyndromic and syndromic PKDs. We also discuss model optimization, success of clinical and preclinical trials, recommendations for the appropriate setup of preclinical trials, and advantages and limitations of these models.

## *In vitro* Models of PKD

A number of *in vitro* experimental models have been used in PKD research, including but not limited to human and animal primary and immortalized renal epithelial, endothelial, and fibroblast cells, and pluripotent stem cells, cultured in two and/or three dimensions (2D: monolayers, and 3D: spheroids and organoids), and *ex vivo* kidney culture (typically embryonic mouse kidneys).^38–48^ In this review, we mainly focus on the *in vitro* PKD model that is the most informative for therapeutic testing, organoids generated from human pluripotent stem cells. Renal epithelial 3D spheroid cultures and *ex vivo* embryonic mouse kidney cultures have also been extensively used for testing the efficacy of compounds aimed to ameliorate or slow PKD cyst formation and growth. However, the mechanism(s) mediating cyst expansion and amelioration in these models seem less clear because large spheres and cysts form and regress in relatively shorter periods, suggesting targeting of secretory mechanisms rather than a combination of mechanisms as observed in PKD. The reader is directed to ref. 48 for a wider review of *in vitro* PKD models.

## Human Kidney Organoids

Organoids are complex tissue-like 3D multicellular *in vitro* structures that recapitulate many but not all the features of the parent organ, allowing for the assessment of kidney features of patients with PKD *in vitro*, and can be generated from embryonic stem cells or induced pluripotent stem cells (iPSCs) by creating the appropriate differentiation conditions.^48^ Indeed, kidney organoids contain both epithelial nephron and supporting cell ultrastructures, providing the most advanced *in vitro* models to date. They are great tools for investigating various cellular features in live cells from the patient tissue context and have been adapted for high-throughput analyses. This approach has advanced greatly over the past ten years, with several studies performed in the PKD field demonstrating that organoids can be generated from iPSCs of patients with ADPKD^49^ or knockout of *PKD1* or *PKD2* in human embryonic stem cells using CRISPR/Cas9.^50^ These systems are amenable to automated high-throughput screens that could be used for disease modeling and drug screening.^41^ Although organoids provide a unique and specialized PKD model, there are a few shortcomings: (*1*) primarily formation of proximal tubules, without collecting ducts, limiting recapitulation of all aspects of ADPKD phenotypes; (*2*) significant variability observed in PKD patient-derived iPSCs for organoid formation and tubule structure, making CRISPR/Cas9-generated models more useful at present; (*3*) lack of critical kidney features, such as vascularization, kidney-specific microenvironment, and fluid flow; and (*4*) the developmental stage of the parent cells dictating organoid maturation.^48,51–53^ To circumvent some of these issues, next-generation organoid models are currently being established, including generation of adult kidney tubular organoids (tubuloids; derived from human kidney tissue and renal cells shed in the urine) and development of organoid-on-a-chip technologies (couple organoids and organ-on-a-chip technology allowing for the addition of fluid flow), which may provide a better model for an adult-onset disease (ADPKD) and one that is more representative of the kidney, respectively.^53–55^

## Animal Models of PKD

A variety of PKD animal model systems have been used in research, including invertebrate models (*Caenorhabditis elegans* and *Drosophila melanogaster*), lower vertebrate models (*Xenopus laevis* and *Danio rerio*), and numerous mammalian models (mice, rats, cats, pigs, horses, and monkeys).^48,56–59^ We have narrowed our focus to animal models that are most frequently used or best suited for drug screening and preclinical testing. For a broader review, see ref. 48

## Zebrafish

**Figure 1.**
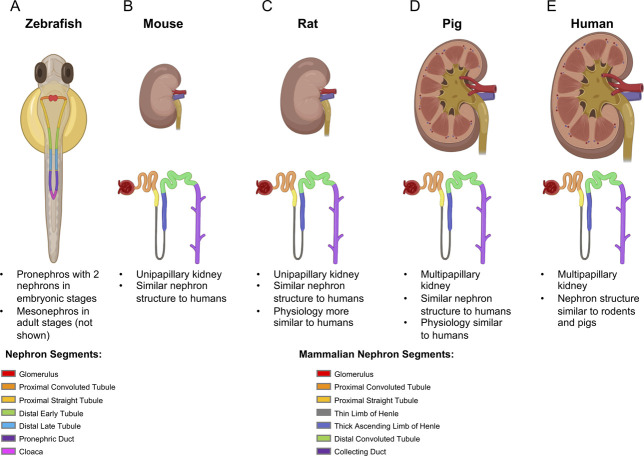
**Kidney and nephron structure across species.** (A) Pronephros and nephron structure in zebrafish and kidney and nephron structure in mice (B), rats (C), pigs (D), and humans (E). Graphics created with Biorender.com.

Although zebrafish (*Danio rerio*) do not have a mammalian-like metanephros, they have a pronephros in embryonic stages (most frequently used) and a mesonephros in adulthood that contains many segments similar to the mammalian kidney (Figure 1). Many zebrafish models have been successfully developed using morpholino injections; however, for the purposes of this review, we focus on stable genetic models generated using transcription activator-like effector nucleases, N-ethyl-N-nitrosourea mutagen, CRISPR/Cas9, or retroviral insertion (Table 1 and Supplemental Table 1). For a broader review, see refs. 48,56.

**Table 1.** Summary of available PKD models *(autosomal dominant polycystic kidney disease only)*

Model				Phenotypes			
	Mutation Mechanism	Human Gene	Disease (Stage)	Kidney	Extrarenal	Survival	Ref(s)
**Zebrafish**							
*pkd1a*^*−/−*^	Knockout, TALENs	*PKD1*	ADPKD (early)	Pronephric cysts	Hydrocephalus; craniofacial defects	ND	^56,60,61^
*pkd2*^−/−^	Knockout, ENU; knockout, retroviral insertion	*PKD2*	ADPKD (early)	No phenotype	Organ laterality defects; laterality defects and body curvature	ND	^56,60,62,63^
**Mice**							
*Pkd1*^nl/nl^	Aberrant splicing (IVS1), reduced PC1 (13%–20%)	*PKD1*	ADPKD (late)	Bilateral cysts and fibrosis	Growth retardation; mild cystic disease in the liver and pancreas; cardiac abnormalities and aortic aneurysms	85% death at 2 mo	^48,64,65^
*Pkd1*^L3/L3^	Aberrant splicing, reduced PC1 (20%–25%)	*PKD1*	ADPKD (late)	Bilateral cysts	Growth retardation	85% death at 6 mo	^65,66^
*Pkd1*^V/V^	Missense mutation in PC1 GPS cleavage site (G-protein coupled receptor proteolytic site; *Pkd1* p.T3041V; *PKD1* p.T3049V)	*PKD1*	ADPKD (late)	Bilateral cysts starting at P1; increased %KW/BW at P4	Reduced BW at P9; bile duct dilatations	50% at P21	^65,67^
*Pkd1*^RC/RC^	Missense mutation mimicked from human patients (p.R3277C); reduced GPS cleavage efficiency and improper folding (approximately 40% mature PC1)	*PKD1*	ADPKD (early and late)	Slowly progressive PKD from birth; increased %KW/BW and BUN at 3 and 9 mo	Mild ductal plate malformations in the liver at 12 mo	Normal	^68,69^
*Pkd1*^RC/−^	Same as above, in combination with a null allele, mimicking biallelic human patients	*PKD1*	ADPKD_VEO_ (late)	Bilateral cysts starting at E16.5; increased %KW/BW at P0; increased BUN at P12	LVH; osteopenia	P28 median	^68^
*Pkd2*^nf3/nf3^	Hypomorphic *Pkd2* model, neomycin cassette with a strong splice acceptor in IVS2; express approximately33% *Pkd2*	*PKD2*	ADPKD (early and late)	Bilateral cysts 6–9 mo (PT)	Growth retardation; pancreatic and bile duct cysts	Viable beyond 12 mo	^65,70^
*Pkd2*^WS25/−^	Exonic insertion of neomycin cassette, resulting in variable endogenous homologous recombination and reduced *Pkd2* expression	*PKD2*	ADPKD (early and late)	Progressive bilateral cysts starting at 1 mo; variable severity	Pancreatic cysts and PLD; variable severity	Reduced after 12 mo	^65,71–73^
**Pigs**							
*PKD1*^+/−^	*PKD1* deletion; *PKD1* expression and translation reduced	*PKD1*	ADPKD (early)	Cysts at 5 mo, begin to deform kidneys at 24 mo	Liver cysts	ND	^48,74,75^

PKD, polycystic kidney disease; TALENs, transcription activator-like effector nucleases; ADPKD, autosomal dominant polycystic kidney disease; ENU, N-ethyl-N-nitrosourea mutagen; ND, not described; IVS, intervening sequence/intron; mo, month; P, postnatal day; %KW/BW, percentage kidney weight/body weight; LVH, left ventricular hypertrophy; VEO, very early-onset; PT, proximal tubule; PLD, polycystic liver disease.

Most zebrafish models develop pronephric cysts, although some do not (*pkd2* and *tsc2*; Table 1 and Supplemental Table 1).^60,62,63^ However, disruption of PKD genes in zebrafish can also lead to obstruction of the cloaca, which results in reduced fluid flow, and increased fluid retention, complicating the interpretation of cystic phenotypes in some cases.^76,77^ Another common feature among zebrafish models of PKD is body or tail curvature, which may be associated with changes in collagen expression in the ADPKD morphants (*pkd1a/b* and *pkd2*).^56,60,78^ Some models also develop laterality (*pkd2*) or liver defects (*tsc2*), both often associated with mutation of PKD/primary cilia genes (Table 1 and Supplemental Table 1).^60,62,63^ Most studies in zebrafish have been performed at embryonic stages; however, some recent studies have evaluated the phenotypes of adult fish (*tmem67*, Supplemental Table 1). There are also differences between morphant and stable knockout/knock-in models for some PKD genes, particularly ADPKD models, where morphants typically exhibit more severe phenotypes (body curvature in *pkd1a/b* morphants and pronephric cysts in *pkd2* morphants), potentially because of maternally contributed transcripts.^56,60,61^

## Rodents

Most of the PKD animal models generated to date have been mouse models because of the shared structural similarity of the mammalian kidney (Figure 1); the availability of numerous tools for generating germline, conditional, and inducible models; and the opportunities for genetic interbreeding (Table 1 and Supplemental Table 1).

**Figure 2.**
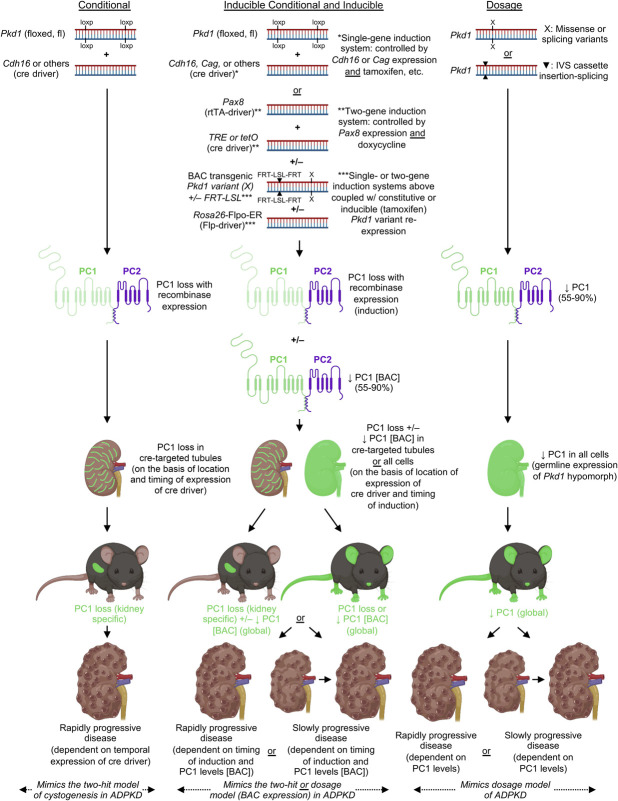
**Conditional, inducible, and dosage mouse models of ADPKD.** Conditional (left panel), inducible conditional and inducible (center panel), and dosage (right panel) mouse models of ADPKD. *Pkd1* models are used as an example. Schematic of genetic manipulations is shown on top, followed by the effect on the protein (polycystin 1 [PC1]), location of the modification in the kidney (green), location within the mice (green), and the resulting renal phenotypes. The BAC system illustrated here is of expression of mutated *Pkd1* in the setting of induced *Pkd1* loss in the kidney.^111^ Re-expression of wild type *Pkd1* on a BAC after induced loss of *Pkd1* can also be used to rescue the cystic phenotype.^102^ Graphics created with Biorender.com. ADPKD, autosomal dominant polycystic kidney disease; rtTA, reverse tetracycline-controlled transactivator; TRE, tetracycline response element; FRT-LSL, loxP-STOP-loxP transcriptional stop sequences flanked by flippase recognition target sequences; BAC, bacterial artificial chromosome.

Focusing initially on germline models, we include only those models that are postnatally viable and, therefore, most suitable for therapeutic testing. Many ADPKD mouse models have been generated for *Pkd1* and *Pkd2*, but because heterozygotes of fully inactivating alleles (modeling the human disease) only develop a few cysts and homozygotes are not viable^71,79^ (also true for rat *Pkd1*^80^), other approaches are required to model the progressive human disease. These approaches can mainly be divided into conditional models (see below) and gene dosage models, which are based on the principle that the level of the functional PKD gene product or protein dictates disease initiation and severity, developed by introducing incompletely penetrant or hypomorphic alleles (see Figure 2 for *Pkd1* examples). Dosage models are often assayed biallelically, resulting in a global reduction but not loss of functional protein, mimicking the haploinsufficiency seen in humans, with the level of the functional PC complex correlated with disease severity (Figure 2 and see Table 1 for examples). Using these approaches, good models of progressive disease, modeling human ADPKD but with more rapid progression, have been developed (including *Pkd1*^L3/L3^, *Pkd1*^RC/RC^, and *Pkd2*^nf3/nf3^; Table 1).^66,68–70^ For ARPKD, complete loss of *Pkhd1* in the mouse results in only mild, late-onset tubule dilatation, although the liver phenotype mirrors the human disease (Supplemental Table 1). A rat model with a spontaneous splicing mutation in *Pkhd1* inducing a frameshift (*Pkhd1*^PCK/PCK^) develops a slowly progressive PKD and PLD phenotype, more like ADPKD than ARPKD (Supplemental Table 1). These studies highlight that there are likely compensatory mechanisms present in rodents, for *Pkhd1*/fibrocystin that do not exist, or are modulated in humans. Because the phenotypes of these models vary so widely (rapid progression versus slowly progressive or mild) and do not always mimic the human disease state, many of these models are most useful for only one particular disease stage (early or late; Table 1).

Many mouse models mimicking syndromic PKDs have also been developed, but global loss of these loci, by definition, often results in a broad range of disease phenotypes (Supplemental Table 1). Because the nonrenal phenotypes can be severe, sometimes resulting in death within the first month of life, or only a limited number of animals survive postnatally, these models can be difficult to use for kidney-related intervention studies (*Anks6*, *Nek8*, *Arl3*, *Cep290*, *Nphp2*, and *Tmem67*; Supplemental Table 1). However, by the deliberate or spontaneous generation of incompletely penetrant alleles, mouse and rat models suitable for therapeutic testing have been developed (*Anks6*^I747N/I747N^, *Nek8*^jck/jck^, and *Nphp3*^pcy/pcy^ mice, and *Tmem67*^wpk/wpk^, *Anks6*^+/Cy^ [Hannover-Sprague-Dawley strain], and *Nek8*^LPK/LPK^ rats; Supplemental Table 1). These and other spontaneous models (*Tg737*^orpk/orpk^ and *Bicc1*^bpk/bpk^ mice and *Pkhd1*^PCK/PCK^ rats) have helped identify disease genes, have been invaluable for studying disease mechanisms, and have been used extensively for preclinical trials.^81–87^ Although the main focus of this review is on ADPKD orthologous disease models, many insights and applications in ADPKD have been obtained through study of these nonorthologous spontaneous models (including disease mechanisms and preclinical studies), highlighting overlap in PKD mechanisms and the value of these models.

**Figure 3.**
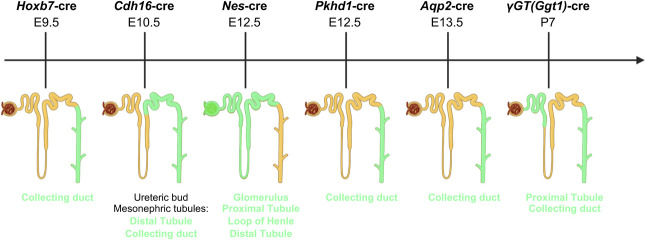
**Cre-driver mouse model expression time line and pattern.** Schematic depicting the temporal and spatial expression of various kidney-specific cre-driver mouse models. Mouse age listed in embryonic days (E) or postnatal days (P). Location of expression within the nephron is depicted and described below in green. Graphics created with Biorender.com.

A more controllable approach to obtain viable models is the use of conditional or inducible mouse models. Because there have been many studies across numerous PKD types, we focus on conditional and inducible ADPKD models as an example (Table 2). However, this approach can be applied to any gene of interest where embryonic or early lethality or severe extrarenal disease is an issue. These models are based on the insertion of targeted sequences into the murine genome that facilitate genetic recombination between two identical sites, induced by a specific recombinase, such as cre or Flp.^105,106^ The control of recombinase expression provides the conditional or inducible nature of these models. We classify these models into three distinct groups: (*1*) conditional, where recombination is controlled by the spatial and temporal expression of a specific gene promoter, including targeting a particular nephron segment at a particular developmental stage/time point (Figure 3); (*2*) conditional inducible, where recombination is spatially controlled by expression of the controlling gene, but temporal expression is induced; and (*3*) inducible, where recombination is spatially broad (because of a more widely expressed promoter), but temporally controlled by an induction agent (Table 2). For ADPKD, these inducible models have been invaluable for illustrating differences in the cystic phenotype depending on when the gene is inactivated. For *Pkd1* models, for example, disruption before the completion of kidney development (<postnatal day [P]14) results in severe, rapidly progressive disease, whereas disruption in >P14 yields a much milder, slowly progressive disease (*Pkd1*^fl/−^ and/or *Pkd1*^fl/fl^, *Cdh16*-cre/ERT2, *Mx1*-cre, and *Cag*-cre/ER; Table 2 and Figure 2).^100,101,104^ These models also neatly mimic the two-hit mechanism of disease that may be important in ADPKD^107–109^ (Figure 2). In one example, a combined dosage and conditional model has been described to generate moderately progressive disease.^110^ A different approach is the use of bacterial artificial chromosome transgenics to express genomic wild-type or mutant *Pkd1* or *Pkd2* and assess gene dosage and the consequences of specific variants in the null context (conditional *Pkd1* or *Pkd2* loss; Figure 2), with incorporation of low copy numbers (typically 1–8).^96,111^ A recent development of this system added an inducible component so that the timing of re-expression of *Pkd1* or *Pkd2* can be analyzed in a time-dependent manner (Figure 2; results discussed later in the *Disease Mechanisms and Genetic Interaction Studies* section^102^). Two separate recombinases (cre and Flp) were used for gene inactivation and reactivation, respectively, and inducible expression of the transgenic *Pkd1* or *Pkd2* allowed phenotypic assessment of wild-type re-expression at particular time points.^102^ This strategy is attractive as an alternative because this allows for spatial and temporal gene inactivation and variant reactivation, and historically, the generation of transgenic models has been more time and cost-effective than the generation of knock-in models.

**Table 2.** Summary of cre-driven mouse models *(autosomal dominant polycystic kidney disease only)*

Model	Mutation Mechanism	Cre driver (Induction Agent/Disease Stage)	Expression Location	Phenotypes	Survival	Ref(s)
**Conditional**						
*Pkd1*^fl/fl^	Deletion of exons 1–4	*Aqp2*-cre (late)	Kidney (collecting ducts) at E13.5; testes and vas deferens	Cystic kidneys and increased kidney weight at 1 wk; severe PKD and increased BUN at 4 wk	Median, approximately 6 wk	^65,88^
*Pkd1*^fl/–^	Deletion of exons 2–6	*γGT(Ggt1)*-cre (late)	Kidney (proximal tubule, collecting duct) at P7; liver and intestine	Progressive and severe PKD from P10 to P26	<1 mo	^65,89,90^
*Pkd1*^fl/fl^	Deletion of exons 2–4	*Hoxb7*-cre (late)	Kidney (collecting ducts) at E9.5; ureter, intestine, and spinal cord	Progressive and severe PKD at P7 and P15; enlarged kidneys (%KW/BW) and increased BUN	ND	^65,91,92^
*Pkd1*^fl/fl^ and *Pkd1*^fl/–^	Deletion of exons 2–4	*Cdh16(Ksp)*-cre (late)	Kidney (ureteric bud, mesonephric tubules) at E10.5 (distal tubules and collecting ducts); Wolffian and Mullerian ducts	Rapidly progressive PKD; enlarged kidneys (%KW/BW) at P4 and increased BUN at P7	P14–P17	^65,93^
*Pkd1*^fl/fl^	Deletion of exons 2–4	*Nes*-cre (late)	Kidney at E12.5 (glomerulus, proximal tubules, loop of Henle, and distal tubules); heart and nervous system	Severe PKD, enlarged kidneys (%KW/BW), increased BUN, and widespread fibrosis at P49	ND	^65,94^
*Pkd1*^fl/–^ and *Pkd1*^fl/fl^	Deletion of exons 2–4	*Pkhd1*-cre (late)	Kidney at E12.5 (collecting ducts); liver	Less severe disease and longer survival than Ksp-cre mice, severe PKD at P24	≥P24	^65,93,95^
*Pkd2*^fl/fl^	Deletion of exons 3–4	*Pkhd1*-cre (late)	Kidney at E12.5 (collecting ducts); liver	Severe PKD at P21	ND	^65,95–97^
*Pkd2*^f3/−^	Deletion of exon 3	*γGT(Ggt1)*-cre (early and late)	Kidney (proximal tubule, collecting duct) at P7; liver and intestine	Kidney cysts at 2 mo	ND	^98,99^
**Conditional inducible**						
*Pkd1*^fl/–^	Deletion of exons 2–11	*Cdh16*-cre/ERT2 (tamoxifen/early and late)	Kidney (collecting duct, loop of Henle, distal tubule)	Postnatal induction (P4), rapid cystic disease after 1 mo; postnatal induction (3–6 mo), mild cystic kidney disease after 3 mo	ND	^65,100^
*Pkd1*^fl/fl^ and *Pkd1*^fl/–^	Deletion of exons 2–6	*Mx1*-cre (pI-pC, IFNa and b dsRNA/early and late)	Kidney; heart, liver, and spleen	Postnatal induction (P7), severe PKD at 7 wk; postnatal induction (5 wk), focal kidney cysts 6–9 wk later, severe PKD and PLD at 13 mo	ND	^65,101^
*Pkd1*^fl/fl^ and *Pkd1*^fl/–^	Deletion of exons 2–4	*Pax8*-rtTA2S*M2 (doxycycline/late)	Kidney (proximal tubule, distal tubule, and collecting duct)	Postnatal induction (P11, 12, and 13), rapid cystic disease at P21	ND	^65,95,102,103^
*Pkd2*^fl/–^	Deletion of exons 3–4	*Pax8*-rtTA2S*M2 (doxycycline/early and late)	Kidney (proximal tubule, distal tubule, and collecting duct)	Postnatal induction (P28–42), severe cystic disease at 16 wk	ND	^102^
**Inducible**						
*Pkd1*^fl/fl^	Deletion of exons 2–4	*Cag*-cre/ER (tamoxifen/early and late)	All tissues	Postnatal induction (P2–P12), severe cystic kidney disease at P19; postnatal induction (P14–6 wk), slowly progressive cystic kidney disease at 6 mo, and liver cysts at 3 mo	ND	^98,104^
*Pkd2*^f3/–^	Deletion of exon 3	*Mx1*-cre (pI-pC, IFNa and b dsRNA/early and late)	All tissues	Postnatal induction (4 wk), kidney, liver, and pancreatic cysts; postnatal induction (6 wk), 50% kidney and pancreatic cysts, 100% liver cysts	ND	^65,99^

E, embryonic day; wk, week; ND, not described; P, postnatal day; PKD, polycystic kidney disease; mo, month; %KW/BW, percentage kidney weight/body weight; dsRNA, double-stranded RNA; BUN, blood urea nitrogen; PLD, polycystic liver disease; rtTA, reverse tetracycline-controlled transactivator.

## Pigs

Pigs have similar renal physiology and structure to humans, with a multipapillary kidney, rather than the unipapillary structure of rodents, and, therefore, represent an excellent kidney disease model (Figure 1).^74^ Unfortunately, the one currently available ADPKD model, a *PKD1*^+/−^ miniature pig model, has slowly progressive PKD with cysts forming at 5 months of age and the kidney structure only beginning to change at 24 months, with some liver cysts (Table 1).^74,75^ Similar to humans and rodents, *PKD1*^−/−^ pigs are lethal. Although mechanistic and preclinical studies have been performed using this model, the slowly progressive nature of the disease and significant cost of housing these larger animals make the existing model complicated for regular use.^74,75^

## Disease Mechanisms and Genetic Interaction Studies

All the PKD models discussed earlier have helped understand PKD mechanisms. Although the pig model is challenging, it has been used to examine glucose metabolism, cell proliferation, increased glycolysis, mammalian target of rapamycin and extracellular signal regulated-kinase signaling, and decreased oxidative phosphorylation and phosphorylation of 5′ adenosine monophosphate-activated protein kinase as potential disease mechanisms.^75^ Interestingly, all these defects and signaling pathways were previously characterized in zebrafish, mouse, and rat models, showing consistency between models.^48^ A large number of other cellular processes have also been implicated using these models, including defective primary cilia and centrosomes; cell cycle abnormalities; protein processing, epigenetic, and autophagy defects; and secretion and immune cell changes. From these studies, changes in a range of signaling pathways have been characterized: Wnt (canonical and noncanonical), STAT6/p100, YAP/yes-associated protein/transcriptional co-activator with PDZ-binding motif), TGF*β*, nuclear factor kappa B, rotein kinase B, hedgehog, cyclic AMP, and calcium.^48,98^ Many of these defects have also been observed in human *in vitro* models; however, differences in cell origin, mechanism of immortalization, and cell line history (cellular characteristics and disease stage, typically end stage) complicate these interpretations.^38^ Expansion of these analyses in kidney organoids, with similar procedures used for model generation, will be greatly beneficial. Furthermore, recapitulating these findings in multiple orthologous models across species will be very helpful in narrowing down relevant cellular defects and disrupted signaling pathways.

Interbreeding of different animal models or genetic interaction studies has also provided valuable information about shared pathways and disease mechanisms. Digenic *Pkd1*; *Pkd2* mouse models have shown an additive effect, in terms of the cystic phenotype, consistent with the dosage of the polycystin-complex being a determinant of disease severity.^112^ Similarly, digenic *Pkd1* and *Pkhd1* studies in mice and rats have shown synergistic enhancement of the renal cystic phenotype, indicating a related function of PC1 and fibrocystin and ultimately providing models that more accurately recapitulate human ARPKD.^80^ The relationship between ADPKD and autosomal dominant PLD was illustrated by enhanced PKD on conditional removal of *Prkcsh* or *Sec63* in the kidney when *Pkd1*^+/−^ or *Pkd2*^+/−^ alleles were present.^96^ These digenic disease enhancements contrast with the reduction in PKD severity associated with removal of primary cilia by conditional loss of *Kif3a* or *Ift20* in kidney tubules, compared with loss of *Pkd1* or *Pkd2* alone.^95,113^ This suggests a PC1-dependent inhibition and cilia-dependent activation pathway that promote cystic growth. Consistent with a polycystin-dependent inhibition pathway, reactivation of *Pkd1* or *Pkd2* transgenic expression after induced loss of the endogenous genes in kidney tubules showed that the cystic phenotype can be halted or even reversed by re-expression of the ADPKD genes.^102^ These results have important implications for future therapies re-expressing the ADPKD gene or repairing the endogenous defect.

## Drug Screening

Because the PKD phenotype is best recapitulated using *in vivo* models, developing systems for moderate to high-level drug screening is a priority for the field. So far, zebrafish models are best suited for this screening, with only low-throughput screening possible in mammals. The body curvature and pronephric cyst phenotypes in zebrafish PKD models have been used for relatively high-throughput drug screening and identifying appropriate doses and toxicity.^48,56,114^ To date, two large drug screening studies have been performed in zebrafish models of PKD, evaluating the efficacy of 115 (histone deacetylase inhibition; *pkd2* and *ift172* models) and 2367 (ALK5 [TGF*β*R1] inhibitors; *pkd2* model) compounds, respectively (Table 3).^63,115^ Although morphants can and have been used for these large screens (histone deacetylase inhibition), use of genetic models is favored because morpholino injections at this scale are typically less practical. Use of zebrafish models for drug screening seems to be an underutilized tool in the field currently. Although kidney organoids do not recapitulate all features of PKD, they do form cysts and potentially provide an opportunity to gather drug screening information from human samples with an individualized medicine approach. High-throughput assays have been developed more recently in this regard but have not yet been used.^41,42^

**Table 3.** Summary of Drug Screening Studies *(zebrafish)*

Model(s)	Gene	Disease	Study Design	Drug/Target Identified	Results	Ref(s)
**Drug screening studies**						
*pkd2*^−/−^ (*hi4166*), *ift172*^−/−^ (*hi2211*), and *pkd2* morphants	*pkd2* and *ift172*	ADPKD, and retinitis pigmentosa and short-rib thoracic dysplasia	Chemical modifier drug screen with a custom library of 115 compounds (cell cycle progression, apoptosis, actin and microtubule cytoskeleton, calcium signaling, vesicular trafficking, receptor tyrosine kinase pathways, posttranslational modifications, protein degradation, and chromatin remodeling)	HDAC inhibition	HDAC inhibition with TSA and VPA corrected body curvature (*pkd2*^−/−^ [*hi4166*]) and reduced cyst formation (*pkd2* morphants) in zebrafish; results verified by treating *Pkd1*^fl/fl^, *Pkhd1*-cre mice with VPA (reduced %KW/BW, CI, and BUN)	^63^
*pkd2*^−/−^ (*hu2173*)	*pkd2*	ADPKD	Unbiased chemical screen using two publicly available compound libraries (spectrum, PKIS; 2367 compounds total)	ALK5 (TGF*β*R1) and noncanonical androgen receptors	Treatment with several steroids, coumarins, and flavonoids (spectrum library) exacerbated PKD phenotypes in *pkd2*^−/−^ zebrafish (tail curvature), and androgen and 5*α*-androstane 3,17-dione had the strongest effect (independent of canonical androgen signaling). Whereas, treatment with several ALK5 (TGF*β*R1) kinase inhibitors (PKIS library; diclofenac, dibutylhydroxyanisole, and zinc pyrithione) partially ameliorated PKD phenotypes (tail curvature) in *pkd2*^−/−^ zebrafish, ultimately validated with the ALK5 inhibitor, SD208. Results verified in 3D cyst cultures	^115^

ADPKD, autosomal dominant polycystic kidney disease; HDAC, histone deacetylase; TSA, trichostatin A, VPA, valproic acid; %KW/BW, percentage kidney weight/body weight; CI, cystic index; BUN, blood urea nitrogen; PKIS, published kinase inhibitor set; PKD, polycystic kidney disease.

## Preclinical Testing: Clinical and Preclinical Trials

Twenty-three different compounds or combinations, targeting approximately 16 different cellular features/pathways, have been or are being tested in clinical trials for the treatment of ADPKD currently (Table 4 and Supplemental Table 2). Many of these are supported by data from preclinical trials performed using many of the animal models described in this review (Table 4). Most of these preclinical trials have been performed in mouse and/or rat models of PKD, both orthologous and nonorthologous; however, pigs (metformin, 5′ adenosine monophosphate-activated protein kinase activator) and zebrafish (metformin; and sirolimus [rapamycin], mTORC1 and 2 inhibitors) have also been used in some cases.^61,74,141^ Although the results of the clinical trials for many of these compounds are not yet available (*n*=15), for the compounds with results (*n*=9), there is some consistency between the findings in humans and animal models *n*=5 but also some differences (*n*=4; Table 4 and Supplemental Table 2). Studies testing the efficacy of pravastatin (HMG-CoA reductase inhibitor), octreotide and pasireotide (somatostatin analogs, growth hormone, insulin, and glucagon inhibitors), bosutinib (Src/Bcr-Abl tyrosine kinase inhibitor), and tolvaptan (vasopressin V2 receptor antagonist; US Food and Drug Administration approved for the treatment of ADPKD) have all shown some consistency between clinical and preclinical trial results, demonstrating improvement in ADPKD phenotypes (Table 4). It should be noted, however, that consistency here is determined solely on the basis of improvement of at least one ADPKD end point (typically total kidney volume for clinical trials), and there are very few compounds that slow the increase in total kidney volume and delay the decline in renal function in human trials. Conversely, studies associated with four compounds have results that are not currently consistent or are not clear between the clinical and preclinical findings, for instance, effective against PLD but not PKD (Table 4). There does not seem to be any correlation between the species or models used and the consistency of results (nonorthologous models have been used in both cases). Interestingly, most of the compounds tested in preclinical trials show consistent results across different species, models, or studies (6/9), with some including three different species and many with two species (Table 4). Human kidney organoids are also starting to emerge as a relevant model for preclinical studies, but current data are limited.^54^ Complementing these *in vitro* studies with *in vivo* studies will still be important, however, to evaluate whole-organ and organismal responses.

**Table 4.** Summary of clinical and preclinical trials *(autosomal dominant polycystic kidney disease; with preclinical results only)*

Intervention	Target/Pathway (PKD Relevance)	Clinical Trials		Preclinical Trials		Clinical/Preclinical Trials Consistent	Preclinical Trials Consistent	Ref(s)
		Number, Status	Results	Species	Results			
Metformin	AMPK activator (energy metabolism sensor; regulates cell growth and proliferation)	2, C	≥50% of the maximal dose safe and well-tolerated (2)	ZF (*pkd2* morphants); MS (*Pkd1*^flox/−^; *Ksp*-cre+ and pCX-creER+); pig (*PKD1*^+/−^)	ZF (↓ cysts, body curvature); MS (↓ CI); pig (↓ KW/BW, TKV, CV, and improved KF)	Unknown: phase 2 only reported	No—three species, four models	ClinicalTrials.gov: NCT02903511, NCT02656017;^74,116–118^
Pravastatin	HMG-CoA reductase inhibitor (lowers lipid levels)	3, C/R	↓ HtTKV	RT (*Anks6*^+/Cy^; Han:SPRD)	Lovastatin ↓ kidney size, CV, and improved KF	Yes	N/A	ClinicalTrials.gov: NCT00456365, NCT03273413, NCT04284657;^119^
RGLS4326 and RGLS8429 (second generation)	microRNA-17 inhibitor (short oligonucleotide; derepresses *PKD1* and *PKD2*)	2, C/R	NA	MS (*Pkd2*^flox/flox^; *Pkhd1*-cre+, *Nek8*^jck/jck^, *Nphp3*^pcy/pcy^, and *Pkd1*^RC/−^)	↓ KW/BW and CI, ↓ KW/BW, and improved KF and survival (RGLS4326)	Unknown	Yes—one species, four models	ClinicalTrials.gov: NCT04536688, NCT05521191; ^86,120^
Sirolimus (rapamycin, rapamune)	mTORC1 and mTORC2/mTOR signaling inhibitor (energy metabolism sensor; regulates cell growth and proliferation)	8, C/U/T	No change in TKV or GFR; ↓ TKV (meta-analysis); ↓ TLV (retrospective transplant study); and NA	ZF (*pkd1a*^−/−^); MS (Tg737^orpk/orpk^ and *Bicc1*^bpk/bpk^); RT (*Anks6*^+/Cy^; Han:SPRD)	ZF (↓ cysts, improved KF); MS (↓ KV, CI; ↓ KW/BW, CI, and improved KF); RT (improved KF, ↓ KW/BW and CV)	Not currently	No—three species, four models	ClinicalTrials.gov: NCT00346918, NCT00286156, NCT01632605, NCT00491517, NCT02055079; NCT01680250; NCT00920309; NCT01223755;^61,83,121–125^
Everolimus	Sirolimus analog; mTORC1/mTOR signaling inhibitor (energy metabolism sensor; regulates cell growth and proliferation)	3, C/T	Slowed TKV increase at study midpoint (not significant at end), but not KF decline; and NA	RT (*Anks6*^+/Cy^; Han:SPRD)	↓ KV, CV, and improved KF (weight gain and KF impaired in treated WT)	Not currently	N/A	ClinicalTrials.gov: NCT00414440; NCT01009957; NCT02134899;^126^
Triptolide	NFkB inhibitor (regulator of proinflammatory and proapoptotic genes)	1, U	NA	MS (*Pkd1*^flox/−^; *Ksp*-cre+ and *Pkd1*^flox/flox^; *Mx1*-cre+); RT (*Anks6*^+/Cy^; Han:SPRD)	MS (↓ KW/BW, CN, CI, and improved KF; and ↓ CN, burden), RT (↓ KW/BW, CV; improved KF)	Unknown	Yes—two species, three models	ClinicalTrials.gov: NCT02115659;^127–129^
Curcumin	Turmeric supplement; NFkB, VEGF, TNF, IL1/6, JAK/STAT, mTOR, AKT, Wnt, cyclooxygenase 2, and five lipoxygenase inhibition (regulators of inflammation, apoptosis, cellular stress, proliferation, *etc.*)	1, C	No reduction in vascular oxidative stress or changes in biomarkers or htTKV	MS (*Pkd1*^flox/flox^; i*Ksp*-cre+)	↓ KW/BW, CA, and improved KF and survival	Not currently	N/A	ClinicalTrials.gov: NCT02494141;^130^
Mesenchymal stem cells	Renotropic, antiapoptotic, antifibrotic, and anti-inflammatory (counteracts apoptosis, fibrosis, and inflammatory programs)	1, C	Safety and tolerability demonstrated	RT (*Pkhd1*^PCK/PCK^)	Improved KF and renal vasculature damage	Unknown	N/A	ClinicalTrials.gov: NCT02166489;^131^
Octreotide (OCT)	Somatostatin (GH-inhibiting hormone) analog; somatostatin receptors, inhibits GH, insulin, and glucagon secretion (targets proliferation and cAMP)	5, C	↓ LV; initially slowed TKV ↑, but not maintained; slowed kidney growth and delayed ESKD onset; and NA	RT (*Pkhd1*^PCK/PCK^)	↓ LW, KW, CV, and fibrosis	PLD, limited for kidney	N/A	ClinicalTrials.gov: NCT03541447; NCT02119052, NCT00309283, NCT01377246, NCT02119013;^132^
Pasireotide (PAS)	High-affinity somatostatin analog (targets proliferation and cAMP)	1, C	Slowed TLV and TKV ↑, no effect on GFR, and ↑ in hyperglycemia and diabetes	MS (*Pkd1*^RC/RC^); RT (*Pkhd1*^PCK/PCK^)	MS (PAS and PAS+tolvaptan: ↓ KW/BW, CV, FV, and LW/BW); RT (PAS and PAS+OCT: ↓ KW/BW, CA, and serum glucose ↑ in PAS but not in PAS+OCT	PLD. limited for kidney, high level of adverse events	Yes—two species, two models	ClinicalTrials.gov: NCT01670110;^133,134^
Bosutinib	Src/Bcr-Abl tyrosine kinase inhibitor (eGFR signaling)	1, C	↓ kidney growth and no change in eGFR	MS (*Bicc1*^bpk/bpk^); RT (*Pkhd1*^PCK/PCK^)	MS (↓ KW/BW, CI, and improved KF); RT (↓ KW/BW, CV, LW/BW, and improved KF)	Yes	Yes—two species, two models	ClinicalTrials.gov: NCT01233869;^84^
Pioglitazone, placebo	Stimulates PPAR*γ* and PPAR*α* (plays a role in cell proliferation, fibrosis, and inflammation)	1, C	Safety demonstrated, but no significant change in TKV or eGFR (pilot study)	MS (*Pkd1*^flox/flox^; iKsp-cre+); RT (*Pkhd1*^PCK/PCK^ and *Tmem67*^wpk/wpk^)	MS (no change in KW/BW, CI, or survival); RT (↓ KW/BW, CV, fibrosis, LW/BW; ↓ KW)	Not clear	No—two species, three models (difference between species)	ClinicalTrials.gov: NCT02697617;^135–137^
KD019 (tesevatinib)	Tyrosine receptor kinase inhibitor (eGFR/ERBB1, HER2/ERBB2, VEGFR, and EphB4 signaling; cell growth)	3, C/T	NA	MS (*Bicc1*^bpk/bpk^); RT (*Pkhd1*^PCK/PCK^)	MS (↓ KW/BW, CI, and improved KF); RT (↓ KW/BW, CI, LW/BW, and improved KF)	Unknown	Yes—two species, two models	ClinicalTrials.gov: NCT01559363, NCT03203642, NCT02616055; ^87^
Tolvaptan (OPC-41061)	Vasopressin V2 receptor antagonist (cAMP regulation, fluid secretion, MAPK/ERK activation/cell proliferation)	21, C/R/U	↓ TKV ↑ and KF decline; adverse effects in all (thirst, pollakiuria, polyuria, and hyperuricemia; 2); different formulations tested; no change in hemodynamics; antagonized L-NMMA effects; improved eGFR sustained; no new safety issues; delayed eGFR decline in patients with very low KF	MS (*Pkd1*^RC/RC^ and *Nphp3*^pcy/pcy^); RT (*Pkhd1*^PCK/PCK^)	MS (↓ KW/BW, CV, and FV; ↓ LnTKV, KW/BW, CV, CN, and improved KF; ↓ KV, KW/BW, CV, and improved survival); RT (↓ KW/BW, CV, and FV; ↓ KW/BW, CI, and improved survival)	Yes	Yes—two species, three models	ClinicalTrials.gov: NCT00541853, NCT00428948, NCT01336972, NCT02527863, NCT02160145, NCT01280721, NCT02251275, NCT01214421, NCT02964273, NCT03764605, NCT03541447, NCT03949894, NCT01022424, NCT02729662, NCT01210560, NCT00841568, NCT01451827, NCT03596957, NCT03803124, NCT02847624, NCT00413777;^69,85,137–140^

C, completed; R, recruiting; U, unknown; T, terminated; ZF, zebrafish; MS, mouse; RT, rat; CI, cystic index; KF, kidney function; TKV, total kidney volume; htTKV, height-adjusted TKV; TLV, total liver volume; NA, not available; N/A, not applicable; cKO, conditional knockout; WT, wild-type; KW/BW, kidney weight/body weight; CV, cystic volume; CN, cyst number; CA, cyst area; FV, fibrotic volume; LW/BW, liver weight/body weight; LnTKV, length-adjusted TKV; GH, growth hormone; cAMP, cyclic AMP; ERK, extracellular signal(en)regulated kinase; mTOR, mammalian target of rapamycin; PLD, polycystic liver disease; PKD, polycystic kidney disease. HMG-CoA, beta-hydroxy beta-methylglutaryl-CoA; VEGF, vascular endothelial growth factor; NFkB, nuclear factor kappa B; AKT, protein kinase B; PPARa, peroxisome proliferator-activated receptors; VEGFR, vascular endothelial growth factor receptor; MAPK, mitogen-activated protein kinase; L-NMMA, nitric oxide synthase inhibitor.

**Figure 4.**
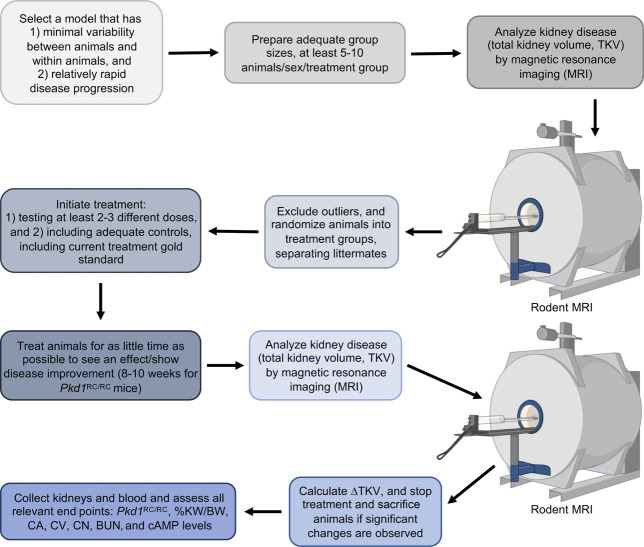
**Suggested preclinical trial workflow *(rodent models)*.** Example workflow and recommendations for preclinical trial setup and execution in rodent models of PKD. Graphics created with Biorender.com. CA, cystic area; cAMP, cyclic AMP; CN, cyst number; BUN, blood urea nitrogen; CV, cystic volume; %KW/BW, percentage kidney weight/body weight; ΔTKV, change in total kidney volume.

Together, these results illustrate the importance of animal models of PKD for preclinical testing, but successful translation from animal models to humans is still challenging. Reasons for failure likely include underpowered studies, different kidney physiology, and toxicity of doses tolerated in rodents versus humans. Therefore, there are important points to consider when setting up and executing preclinical trials, and we include some suggestions on the design and execution of preclinical trials in the workflow shown in Figure 4. For preclinical trials in ADPKD, performing studies in both a conditional and dosage model and using models that do not have too rapidly progressive disease (mimicking the human disease) are approaches that the field should adopt. These standards have mainly been developed for rodent models, so additional considerations and modifications will be required for other species.^69^

## Advantages and Limitations

There are advantages and limitations for each of the experimental PKD animal models described above (Table 5). Human kidney organoids are the most advanced, established, *in vitro* model currently available and are beneficial because they allow for the assessment of diseased human kidney cell features in live cells, use of individualized medicine patient and variant-specific contexts, and are amenable to high-throughput analyses. They unfortunately lack several key aspects of kidneys, however, including collecting ducts (proximal tubules primarily form), vasculature, a kidney-specific microenvironment, and fluid flow (Table 5). Use of zebrafish is advantageous because they are amenable to high-throughput analyses; are transparent and have *ex utero* development; and are small, allowing easy handling and low maintenance costs. However, they are limited in that they have many duplicated genes; there is not a complete assessment of organ effects; and cloaca obstruction in some PKD models complicates the interpretation of cystic phenotypes (Table 5). Mice have a mammalian kidney structure; there are numerous models/tools available; they have a short gestation period (21 days); and maintenance and housing are relatively easy. However, there are physiological and structural (mainly associated with a simplified papillary and nephron branching structure compared with humans; higher order branching and associated molecular signatures in humans and rhesus monkeys described here^142^) kidney changes between mice and humans, and a significant proportion of therapies have not translated to humans (Table 5).^94,121,143^ Rats have renal physiology more similar to humans and are still relatively easy to house and maintain. However, there are still structural changes in the kidney compared with humans (similar to mice); fewer models/tools are available, given the difficulty with genetic manipulations in the past; and they require a larger housing footprint (Table 5). Pigs have the most similar kidney physiology and structure to humans; treatment doses should be quite similar to humans; and they have been a useful model for other diseases. Unfortunately, they have a longer gestation period and time to sexual maturity (114 days and 5–6 months); housing requires a larger space and involves higher cost; good models for preclinical trials have not yet been developed; and therapeutic testing would be very costly (Table 5).

**Table 5.** Model advantages and limitations

Model System	Disease Mechanisms	Drug Screening	Preclinical Testing	Advantages	Limitations
Human kidney organoids	Yes	Yes	Yes	∙ Form complex tissue-like 3D multicellular *in vitro* structures that recapitulate many disease features and form nephron epithelial and supporting cell ultrastructures ∙ Allows for assessment of diseased human kidney features in live cells ∙ Individualized medicine approaches could potentially be applied in a patient/variant-specific context ∙ Does not require a large footprint or specialized animal equipment/husbandry ∙ Amenable to high-throughput analyses	∙ Primarily form proximal tubules, without collecting tubules, limiting disease recapitulation ∙ Significant variability has been observed in patient-derived iPSCs (organoid formation and tubule structure) ∙ Lack of critical kidney features (vascularization, kidney-specific microenvironment, and fluid flow) ∙ Developmental stage of parent cells dictates organoid maturation ∙ Short experimental duration (viable for <1 wk)
Zebrafish	Yes	Yes	Yes	∙ High number of offspring ∙ High-throughput analysis (early developmental stages) ∙ Simple and cost-effective genetic manipulations ∙ Transparency and ex utero development ∙ Small size (easy handling and low cost) ∙ Clear phenotypic assays available ∙ Major drug classes behave similarly in fish and humans ∙ Useful for drug toxicity studies (functional liver at 72 hpf)	∙ Many duplicated genes ∙ Only early stages can be used for high-throughput screens ∙ Pronephros only has motile cilia ∙ Embryo chorion can impair drug penetration and must be removed (48–72 hpf) ∙ Difficult to determine drug doses absorbed ∙ Incomplete assessment of organ effects (lung and mammary glands) ∙ Cloaca obstruction in some PKD models complicates phenotypic interpretation
Mice	Yes	No	Yes	∙ Kidney structure more similar to humans ∙ Numerous models/tools available for various disease types/applications ∙ Short gestation period ∙ Relatively easy maintenance and housing ∙ Phenotypic variation across different genetic backgrounds^a^	∙ Physiological and kidney structural changes between mice and humans ∙ Many therapies have not translated to humans, but use of nonorthologous models likely complicates issue ∙ Phenotypic variation across different genetic backgrounds^a^
Rats	Yes	No	Yes	∙ Physiology more similar to humans ∙ Still relatively easy to house and manage ∙ Phenotypic variation across different genetic backgrounds^a^	∙ Fewer models/tools available, given difficulty with genetic manipulations in the past ∙ Larger housing footprint required, and fewer animals per cage ∙ Phenotypic variation across different genetic backgrounds^a^
Pigs	Yes	No	Yes	∙ Physiology and kidney structure similar to humans ∙ Treatments and doses should be quite similar in humans ∙ Strong sequence similarity with humans (*PKD1* and *PKD2*, ADPKD) ∙ Has been a useful model for other diseases	∙ Longer gestation and time to sexual maturity ∙ Housing requires a larger space and involves higher cost ∙ Animal handling is more challenging, given large size of some breeds ∙ Preclinical trials would need lower numbers and would be very costly

^a^Considered as an advantage and limitation; background strain effects can be advantageous for development of models with or without particular disease features, but can also be a limitation in that results can be variable dependent on the strain(s) used in studies.

iPSC, induced pluripotent stem cell; hpf, hours post fertilization; PKD, polycystic kidney disease; ADPKD, autosomal dominant polycystic kidney disease.

## Conclusion

Overall, all the experimental models discussed in this review have value in several different areas, and each have advantages, but also have limitations. When selecting a model for your studies, the application and resources available are likely the most important things to consider. In addition, using multiple models is always a good strategy, whether it is multiple animal models or a combination of animal and *in vitro* models. Organoids and next-generation models (tubuloids and organoid on a chip), as discussed earlier, have shown great advances in recent years,^54,55,144,145^ and a combination of animal and human models for preclinical and other studies would be of great value and will hopefully aid in translation of novel therapies into clinical trials and human patients—a major hurdle in the past.

Although the development of the animal models described earlier has led to countless important findings in the PKD field, there are still some gaps that we should consider filling. These include identifying and generating improved animal models for ARPKD, expanding our repertoire of rat models, and considering ways to improve and expand the currently available pig models. Carefully considering the design and execution of preclinical trials, using multiple models, if possible, should also be considered as an important goal to improve the translation of these trials to humans.

## Supplementary Material

SUPPLEMENTARY MATERIAL

## Supplemental Material

Supplemental Table 1. Summary of available PKD models (ARPKD and syndromic PKDs only).

Supplemental Table 2. Summary of clinical and preclinical trials (ADPKD only; no preclinical results).

Supplemental References.
