# Supplementary material for: Experimental Models of Polycystic Kidney Disease: Applications and Therapeutic Testing
Source: Kidney360. 2023 Jul 7;4(8):1155–73. doi: 10.34067/KID.0000000000000209 (PMC10476690; doi:10.34067/KID.0000000000000209)
Supplement: SUPPLEMENTARY MATERIAL [file kidney360-4-1155-s001.pdf]

Experimental Models of Polycystic Kidney Disease: Applications and Therapeutic Testing

Cynthia J. Sieben, Ph.D.[1] and Peter C. Harris, Ph.D.[1]

[1]Division of Nephrology and Hypertension, Mayo Clinic, Rochester, MN

Supplemental Information

Table of Contents

Supplemental Table 1. Summary of Available PKD Models *(ARPKD and syndromic PKDs only)* 3

Supplemental Table 2. Summary of Clinical and Preclinical Trials *(ADPKD only; no preclinical results)* 8

Supplemental References 10

Supplemental Table 1. Summary of Available PKD Models *(ARPKD and syndromic PKDs only)*

Model	Mutation mechanism	Human Gene	Disease (stage)	Phenotypes: Kidney	Phenotypes: Extrarenal	Phenotypes: Survival	Ref(s)
Zebrafish							
dzip1l$^{-/-}$	Knockout, CRISPR	*DZIP1L*	ARPKD (Early?)	Pronephric cysts	Body curvature, hydrocephalus, and otolith defects	ND	[1,2]
cc2d2a$^{-/-}$	Konckout, ENU	*CC2D2A*	JBTS (Early?)	Pronephric cysts	Pericardial edema, and body curvature	ND	[2,3]
inpp5e$^{-/-}$	Knockout, CRISPR	*INPP5E*	JBTS (Early?)	Pronephric cysts	Pericardial effusion, and body curvature	ND	[2,4,5]
arl13b$^{-/-}$	Knockout, retroviral insertion	*ARL13B*	JBTS (Early?)	Pronephric cysts	Body curvature	ND	[2,6]
tmem67$^{e3/e3}$	Hypomorphic, TALENs (~10% TMEM67)	*TMEM67*	MKS, JBTS, NPHP, RHYNS, CHF (Early?)	Pronephric and mesonephric cysts; more prominent in adult male fish	Body curvature in embryos and adults	Viable to at least 15m	[7]
ift172$^{-/-}$	Knockout, retroviral insertion	*IFT172*	Short-rib thoracic dysplasia (Early?)	Renal cysts	Body curvature, and cartilage defects	ND	[2,8-10]
tsc2$^{-/-}$	Knockout, ENU	*TSC2*	TSC (Early?)	No phenotype	Brain defects and enlarged liver	Increased embryonic mortality	[2,11]
Mice							
Pkhd1$^{del2/del2}$	Knockout, deletion of exon 2	*PKHD1*	ARPKD (Early)	Dilation of PT at advanced age; phenotypes more severe in females?	Severe PLD; occasional pancreatic cysts; phenotypes more severe in females?	ND	[12,13]
Pkhd1$^{lsl/lsl}$	Knockout, insertion of lox-stop-lox (lsl) cassette in IVS2	*PKHD1*	ARPKD (Early)	Dilation of PT at advanced age; phenotypes more severe in females?	Severe PLD; occasional pancreatic cysts; phenotypes more severe in females?	ND	[13,14]

Pkhd1$^{C642X/+}$	Truncation, C642X (c.1926_1932delTGATTGG, in exon 20)	*PKHD1*	ARPKD (Early)	Dilation of PT at advanced age (1.5y); phenotypes more severe in females?	Biliary cysts and increased number of bile ducts	ND	[13,15]
Pkhd1$^{del3-4/del3-4}$	Knockout, deletion of exons 3-4	*PKHD1*	ARPKD (Early)	Dilation of CD and TALH (6m or older, ~55%)	Abnormalities of the biliary tract; subset of animals exhibit pancreatic cysts, perinatal respiratory failure, or growth retardation	ND	[13,16]
Pkhd1$^{lacZ/lacZ}$	Knockout, replacement of exons 1-3 with a LacZ reporter gene	*PKHD1*	ARPKD (Early)	Dilation of PT, CD, and glomeruli at 9m	Dilatation of the bile ducts and periportal fibrosis; dilatation of the pancreatic exocrine ducts, with less frequent pancreatic cysts	ND	[13,17]
Pkhd1$^{del15-16\ GFP/del15-16\ GFP}$	Knockout, deletion of exon 16 and disruption of exon 15 by insertion of GFP/Neomycin cassette	*PKHD1*	ARPKD (Early)	Mild to severe tubule dilation or cyst formation (~10% early onset [4m] and ~60% late onset [after 1 yr]	Cysts, fibrosis, and necrosis in the liver (4m); dilation of pancreatic ducts with interstitial fibrosis (6m)	Median ~10m	[13,18]
Bbs2$^{–/–}$	Knockout, deletion of exons 5-14	*BBS2*	BBS (Early)	Inflammatory infiltration and multiple cysts (5m)	Obesity; retinal degeneration; accumulation of lipids in the liver; male infertility	ND	[19]
Bbs4$^{–/–}$	Knockout, gene trap IVS1	*BBS4*	BBS (Early)	Tubular cystic lesions (6m); inflammatory infiltration; later onset of glomerular cysts	Obesity; metabolic syndrome; anosmia; male infertility; hydrometrocolpos; neural tube defects; retinal degeneration; hypertension; perinatal lethality	ND	[20,21]
Bbs8$^{–/–}$	Knockout, deletion of exons 1-2	*BBS8*	BBS (Early)	Mild dilation (8m)	Reduced olfactory response	ND	[22]

*Cys1*cpk/cpk	Spontaneous deletion of 2 portions of exon 1, premature stop codon	*CYS1*	Boichis disease (Early & Late)	Bilateral cysts	Liver inflammation; pancreatic cysts	3-4w	[13,23,24]
*Bicc1*bpk/bpk	Spontaneous insertion of 2 bases in exon 22	*BICC1*	CYSRD (Early & Late)	Bilateral cysts	Liver cysts	ND	[13,25-29]
Bicc1jcpk/jcpk	Splicing mutation, premature stop codon	*BICC1*	CYSRD (Early & Late)	Bilateral cysts	Bile duct defects	ND	[13,27,30-35]
*Arl3*Gt(neo)1Lex/Gt(neo)1Lex	Aberrant splicing (IVS1 insertion of neomycin cassette)	*ARL3*	JBTS (Late)	Defective development and cysts	Bile duct and gall bladder defects; pancreatic cysts	Maximum P21	[13,36]
Tmem218–/–	Knockout, gene trap IVS1	*TMEM218*	JBTS, MKS (Early & Late)	Bilateral cysts, inflammation, and fibrosis (14w)	Retinal degeneration	ND	[37]
Cep290–/–	Knockout, deletion of exons 1-4	*CEP290*	MKS, JBTS, NPHP, BBS (Late)	Bilateral cysts (12m- small number of survivors without hydrocephalus)	Early vision loss; hydrocephalus	80% death by P21	[38]
*Cep290*Gt/Gt	Truncation, gene trap IVS25	*CEP290*	MKS, JBTS, NPHP, BBS (Late)	Severe cystic disease (P19)	Majority of mice die mid-gestation; occasional survivors have hydrocephalus and severe cystic kidneys	E13-14	[38]
*Tmem67*Bpck/Bpck	Knockout, large deletion including *Tmem67* and 5 other genes	*TMEM67*	MKS, JBTS, NPHP, RHYNS, CHF (Late)	Bilateral cysts (E16.5)	Retinal degeneration; skeletal defects; cochlea defects; occasional hydrocephalus	P16/P13	[39,40]
Tmem67–/–	Knockout, deletion of exons 2-3	*TMEM67*	MKS, JBTS, NPHP, RHYNS, CHF (Late)	Bilateral cysts (E18.5)	Early postnatal lethality	ND	[41]
*Anks6*I747N/I747N	Missense mutation (p.I747N; induced by ENU)	*ANKS6*	NPHP (Early & Late)	Slowly progressive cyst formation from birth to 12m (peak %KW/BW and renal dysfunction)	None described	18m	[42]

Anks6$^{Strkr/Strkr}$	Missense mutation (p.M187K; induced by ENU)	*ANKS6*	NPHP (Early & Late)	Glomerular cysts (at birth) and cystic dilatation (first several wks); inflammation (9-10m)	Heterotaxy and *situs inversus*; cardiopulmonary malformations	Perinatal lethality in mice with CHD; those without CHD survive long term (evaluated up to 10m)	43
Nek8$^{jck/jck}$	Spontaneous missense mutation (p.G448V)	*NEK8*	NPHP, Renal-hepatic-pancreatic dysplasia 2 (Early & Late)	Enlarged kidneys with cysts at 4w	No other abnormalities described	~20-25w	13,44-46
Nek8$^{Roc/Roc}$	Missense mutation (p.I124T; induced by ENU)	*NEK8*	NPHP, Renal-hepatic-pancreatic dysplasia 2 (Early & Late)	Glomerular cysts (at birth) and cystic dilatation (first several wks); occasional hydroureter, hyrdonephrosis, and duplex kidneys	Heterotaxy and *situs inversus*; cardiopulmonary malformations	Perinatal lethality in mice with CHD; those without CHD are postnatally viable	43
Nphp2$^{-/-}$	Knockout, deletion exons 3-11 (transgenic insertion)	*NPHP2*	NPHP (Late)	Bilateral cysts (E15)	Altered left-right laterality; bile duct malformations; postnatal death	P7	47
Nphp3$^{pcy/pcy}$	Spontaneous missense mutation (p.I1614S)	*NPHP3*	NPHP (Early & Late)	Inflammation, fibrosis, and cysts	Occasional intracranial aneurysm; craniofacial and skeletal defects	~40w, but 2y in C57BL6 background	13,48,49
Nek1$^{kat\text{-}2J/kat\text{-}2J}$	Spontaneous insertion (c.966insG), resulting a frameshift and premature stop	*NEK1*	Short rib-polydactyly syndrome type II (Early & Late)	Progressive bilateral cysts (3m)	Runted; hydrocephalus; anemia; nervous system and craniofacial defects; male sterility; portal bile duct dilation in older mice	Median 211d	13,50,51
Rats							

Pkhd1$^{PCK/PCK}$	Spontaneous missense mutation in IVS35, resulting in frameshift	*PKHD1*	ARPKD (Early & Late)	Progressive PKD starting from 20d; more severe in males	Progressive PLD, more prominent in females	18m	13,52,53
Tmem67$^{wpk/wpk}$	Spontaneous missense mutation (p.P394L)	*TMEM67*	MKS, JBTS, NPHP, RHYNS, CHF (Late)	Severe cystic disease, enlargement, and uremia at P21	Brain, retinal, and fertility defects (males)	ND	13,54-57
Anks6$^{+/Cy}$ (Han:SPRD)	Spontaneous missense mutation (p.R823W)	*ANKS6*	NPHP (Early & Late)	Progressive PKD, fibrosis, inflammation; uremia and proteinuria in males after 10m	Liver and pancreatic cysts in females after 17m; hyperlipidemia and hypertension in males after 10m	12-18m *Anks6*$^{+/Cy}$ (3w in *Anks6*$^{Cy/Cy}$)	13,58-61
Nek8$^{LPK/LPK}$	Spontaneous missense mutation (p.R650C)	*NEK8*	NPHP, Renal-hepatic-pancreatic dysplasia 2 (Early & Late?)	Cyst formation	Hypertension	ND	13,62,63

ARPKD, autosomal recessive polycystic kidney disease; ND, not determined; TSC, tuburous sclerosis complex; MKS, meckel syndrome; JBTS, joubert syndrome; NPHP, nephronophthisis; RHYNS, retinitis pigmentosa-hypopituitarism-nephronophthisis-skeletal dysplasia syndrome; CHF, congenital hepatic fibrosis; VEO, very early-onset; CYSRD: cystic renal dysplasia; BBS, bardet-biedl syndrome

Supplemental Table 2. Summary of Clinical and Preclinical Trials *(ADPKD only; no preclinical results)*

Intervention	Target/Pathway (PKD Relevance)	Clinical Trials: Number, Status	Clinical Trials: Results	Preclinical Trials: Species	Preclinical Trials: Results	Clinical/ Preclinical Trials Consistent	Preclinical Trials Consistent	Ref(s)
Lisinopril, Telmisartan, placebo	ACEI, ARB (inhibits ACE & ARB, lowers BP)	2, C	ACEI controlled BP; ARB did not impact eGFR	N/A	N/A	Unknown	N/A	ClinicalTrials.gov: NCT01885559, NCT00283686
Candesartan, Clinidipine, and non-calcium channel blocker (CCB) agents	ARB, CCB, and other antihypertensive drugs (inhibits ARB and voltage-dependent calcium channels, lowers BP)	1, U	NA	N/A	N/A	Unknown	N/A	ClinicalTrials.gov: NCT00541853
Clinidipine, Imidapril	CCB, ACEI (inhibits voltage-dependent calcium channels and ACE, lowers BP)	1, U	NA	N/A	N/A	Unknown	N/A	ClinicalTrials.gov: NCT00890279
Tetracosactin	Adrenocorticotropic hormone (ACTH) peptide (increases aldosterone, counteracting hyponatremia, hyperkalemia, and metabolic acidosis)	1, C	NA	N/A	N/A	Unknown	N/A	ClinicalTrials.gov: NCT00598377
Spironolactone	Aldosterone Receptor Antagonist (endothelial cell dysfunction & arterial stiffness)	1, C	Vascular endothelial cell function not improved, despite ↓ BP	N/A	N/A	Unknown	N/A	ClinicalTrials.gov: NCT01853553
AL01211	Glucosylceramide Synthase (GCS) Inhibitor (reduces glycosphingolipid formation; regulate proliferation, apoptosis, and cell signaling)	1, R	NA	NA	NA	Unknown	NA	ClinicalTrials.gov: NCT04908462

Bardoxolone	Nrf2 pathway activator/NFkB inhibitor (activates Nrf2 antioxidant pathway & inhibits pro-inflammatory & pro-apoptotic NFkB pathway)	3, C/R	NA	N/A	N/A	Unknown	N/A	ClinicalTrials.gov: NCT03918447, NCT03749447, NCT03366337; [64]
Ng-monomethyl-L-arginine (L-NMMA)	Nitric oxide (NO) synthase inhibitor (inhibits natriuresis & diuresis)	1, C	NA	N/A	N/A	Unknown	N/A	ClinicalTrials.gov: NCT00345137
Lanreotide	Somatostatin (growth hormone [GH] inhibiting hormone) analogue; Somatostatin receptors, inhibits GH, insulin, & glucagon secretion (targets proliferation & cAMP)	4, C/U	↓ TKV, TLV, no improvement in KF, & ↑ incidence of gallstones; ↓ TKV, TLV, & eGFR only after start; & NA	N/A	N/A	Unknown	N/A	ClinicalTrials.gov: NCT01616927, NCT01354405, NCT02127437, NCT00565097

ACEI, ACE inhibitor; ARB, Angiotensin Receptor Blocker; BP, blood pressure; eGFR, estimated GFR; C, Completed; R, Recruiting; U, Unknown; NA, not available; N/A, not applicable; TKV, total kidney volume; TLV, total liver volume; KF, kidney function

Supplemental References

1. Lu, H., Galeano, M.C.R., Ott, E., Kaeslin, G., Kausalya, P.J., Kramer, C., Ortiz-Bruchle, N., Hilger, N., Metzis, V., Hiersche, M., et al. (2017). Mutations in DZIP1L, which encodes a ciliary-transition-zone protein, cause autosomal recessive polycystic kidney disease. Nat Genet *49*, 1025-1034. 10.1038/ng.3871.
2. Elmonem, M.A., Berlingerio, S.P., van den Heuvel, L.P., de Witte, P.A., Lowe, M., and Levtchenko, E.N. (2018). Genetic Renal Diseases: The Emerging Role of Zebrafish Models. Cells *7*. 10.3390/cells7090130.
3. Gorden, N.T., Arts, H.H., Parisi, M.A., Coene, K.L., Letteboer, S.J., van Beersum, S.E., Mans, D.A., Hikida, A., Eckert, M., Knutzen, D., et al. (2008). *CC2D2A* is mutated in Joubert Syndrome and interacts with the ciliopathy-associated basal body protein CEP290. Am J Hum Genet *83*, 559-571.
4. Luo, N., Lu, J., and Sun, Y. (2012). Evidence of a role of inositol polyphosphate 5-phosphatase INPP5E in cilia formation in zebrafish. Vision Res *75*, 98-107. 10.1016/j.visres.2012.09.011.
5. Xu, W., Jin, M., Hu, R., Wang, H., Zhang, F., Yuan, S., and Cao, Y. (2017). The Joubert Syndrome Protein Inpp5e Controls Ciliogenesis by Regulating Phosphoinositides at the Apical Membrane. J Am Soc Nephrol *28*, 118-129. 10.1681/asn.2015080906.
6. Cantagrel, V., Silhavy, J.L., Bielas, S.L., Swistun, D., Marsh, S.E., Bertrand, J.Y., Audollent, S., Attié-Bitach, T., Holden, K.R., Dobyns, W.B., et al. (2008). Mutations in the cilia gene ARL13B lead to the classical form of Joubert syndrome. Am J Hum Genet *83*, 170-179. 10.1016/j.ajhg.2008.06.023.
7. Zhu, P., Qiu, Q., Harris, P.C., Xu, X., and Lin, X. (2021). mtor Haploinsufficiency Ameliorates Renal Cysts and Cilia Abnormality in Adult Zebrafish tmem67 Mutants. J Am Soc Nephrol *32*, 822-836. 10.1681/asn.2020070991.
8. Halbritter, J., Bizet, A.A., Schmidts, M., Porath, J.D., Braun, D.A., Gee, H.Y., McInerney-Leo, A.M., Krug, P., Filhol, E., Davis, E.E., et al. (2013). Defects in the IFT-B component IFT172 cause Jeune and Mainzer-Saldino syndromes in humans. Am J Hum Genet *93*, 915-925. 10.1016/j.ajhg.2013.09.012.
9. Lunt, S.C., Haynes, T., and Perkins, B.D. (2009). Zebrafish *ift57, ift88,* and *ift172* intraflagellar transport mutants disrupt cilia but do not affect hedgehog signaling. Dev Dyn *238*, 1744-1759. 10.1002/dvdy.21999.
10. Cao, Y., Semanchik, N., Lee, S.H., Somlo, S., Barbano, P.E., Coifman, R., and Sun, Z. (2009). Chemical modifier screen identifies HDAC inhibitors as suppressors of PKD models. Proc Natl Acad Sci U S A *106*, 21819-21824. 10.1073/pnas.0911987106.
11. Kim, S.H., Speirs, C.K., Solnica-Krezel, L., and Ess, K.C. (2011). Zebrafish model of tuberous sclerosis complex reveals cell-autonomous and non-cell-autonomous functions of mutant tuberin. Dis Model Mech *4*, 255-267. 10.1242/dmm.005587.
12. Woollard, J.R., Punyashtiti, R., Richardson, S., Masyuk, T.V., Whelan, S., Huang, B.Q., Lager, D.J., vanDeursen, J., Torres, V.E., Gattone, V.H., et al. (2007). A mouse model of autosomal recessive polycystic kidney disease with biliary duct and proximal tubule dilatation. Kidney Int *72*, 328-336. 10.1038/sj.ki.5002294.
13. Holditch, S.J., Nemenoff, R.A., and Hopp, K. (2020). In Polycystic Kidney Disease, (CRC Press), pp. 193-243.
14. Bakeberg, J.L., Tammachote, R., Woollard, J.R., Hogan, M.C., Tuan, H.F., Li, M., van Deursen, J.M., Wu, Y., Huang, B.Q., Torres, V.E., et al. (2011). Epitope-tagged Pkhd1 tracks the processing, secretion, and localization of fibrocystin. J Am Soc Nephrol *22*, 2266-2277. 10.1681/ASN.2010111173.
15. Shan, D., Rezonzew, G., Mullen, S., Roye, R., Zhou, J., Chumley, P., Revell, D.Z., Challa, A., Kim, H., Lockhart, M.E., et al. (2019). Heterozygous Pkhd1(C642*) mice develop cystic

liver disease and proximal tubule ectasia that mimics radiographic signs of medullary sponge kidney. Am J Physiol Renal Physiol *316*, F463-F472. 10.1152/ajprenal.00181.2018.

16. Garcia-Gonzalez, M.A., Menezes, L.F., Piontek, K.B., Kaimori, J., Huso, D.L., Watnick, T., Onuchic, L.F., Guay-Woodford, L.M., and Germino, G.G. (2007). Genetic interaction studies link autosomal dominant and recessive polycystic kidney disease in a common pathway. Hum Mol Genet *16*, 1940-1950. 10.1093/hmg/ddm141.
17. Williams, S.S., Cobo-Stark, P., James, L.R., Somlo, S., and Igarashi, P. (2008). Kidney cysts, pancreatic cysts, and biliary disease in a mouse model of autosomal recessive polycystic kidney disease. Pediatr Nephrol *23*, 733-741. 10.1007/s00467-007-0735-4.
18. Kim, I., Fu, Y., Hui, K., Moeckel, G., Mai, W., Li, C., Liang, D., Zhao, P., Ma, J., Chen, X.Z., et al. (2008). Fibrocystin/polyductin modulates renal tubular formation by regulating polycystin-2 expression and function. J Am Soc Nephrol *19*, 455-468. 10.1681/ASN.2007070770.
19. Nishimura, D.Y., Fath, M., Mullins, R.F., Searby, C., Andrews, M., Davis, R., Andorf, J.L., Mykytyn, K., Swiderski, R.E., Yang, B., et al. (2004). *Bbs2*-null mice have neurosensory deficits, a defect in social dominance, and retinopathy associated with mislocalization of rhodopsin. Proc Natl Acad Sci U S A *101*, 16588-16593. 10.1073/pnas.0405496101.
20. Eichers, E.R., Abd-El-Barr, M.M., Paylor, R., Lewis, R.A., Bi, W., Lin, X., Meehan, T.P., Stockton, D.W., Wu, S.M., Lindsay, E., et al. (2006). Phenotypic characterization of Bbs4 null mice reveals age-dependent penetrance and variable expressivity. Hum Genet *120*, 211-226. 10.1007/s00439-006-0197-y.
21. Guo, D.F., Beyer, A.M., Yang, B., Nishimura, D.Y., Sheffield, V.C., and Rahmouni, K. (2011). Inactivation of Bardet-Biedl syndrome genes causes kidney defects. Am J Physiol Renal Physiol *300*, F574-580. 10.1152/ajprenal.00150.2010.
22. Tadenev, A.L., Kulaga, H.M., May-Simera, H.L., Kelley, M.W., Katsanis, N., and Reed, R.R. (2011). Loss of Bardet-Biedl syndrome protein-8 (BBS8) perturbs olfactory function, protein localization, and axon targeting. Proc Natl Acad Sci U S A *108*, 10320-10325. 10.1073/pnas.1016531108.
23. Hou, X.Y., Mrug, M., Yoder, B.K., Lefkowitz, E.J., Kremmidiotis, G., D'Eustachio, P., Beier, D.R., and Guay-Woodford, L.M. (2002). Cystin, a novel cilia-associated protein, is disrupted in the cpk mouse model of polycystic kidney disease. Journal of Clinical Investigation *109*, 533-540. 10.1172/Jc1200214099.
24. Chiu, M.G., Johnson, T.M., Woolf, A.S., Dahm-Vicker, E.M., Long, D.A., Guay-Woodford, L., Hillman, K.A., Bawumia, S., Venner, K., Hughes, R.C., et al. (2006). Galectin-3 associates with the primary cilium and modulates cyst growth in congenital polycystic kidney disease. Am J Pathol *169*, 1925-1938. 10.2353/ajpath.2006.060245.
25. Talbot, J.J., Shillingford, J.M., Vasanth, S., Doerr, N., Mukherjee, S., Kinter, M.T., Watnick, T., and Weimbs, T. (2011). Polycystin-1 regulates STAT activity by a dual mechanism. Proc Natl Acad Sci U S A *108*, 7985-7990. 10.1073/pnas.1103816108.
26. Nuovo, G.J., MacConnell, P., Forde, A., and Delvenne, P. (1991). Detection of human papillomavirus DNA in formalin-fixed tissues by in situ hybridization after amplification by polymerase chain reaction. Am J Pathol *139*, 847-854.
27. MacRae Dell, K., Nemo, R., Sweeney, W.E., Jr., and Avner, E.D. (2004). EGF-related growth factors in the pathogenesis of murine ARPKD. Kidney Int *65*, 2018-2029. 10.1111/j.1523-1755.2004.00623.x.
28. Veizis, E.I., Carlin, C.R., and Cotton, C.U. (2004). Decreased amiloride-sensitive Na+ absorption in collecting duct principal cells isolated from BPK ARPKD mice. Am J Physiol Renal Physiol *286*, F244-254. 10.1152/ajprenal.00169.2003.
29. Ozawa, Y., Nauta, J., Sweeney, W.E., and Avner, E.D. (1993). A new murine model of autosomal recessive polycystic kidney disease. Nihon Jinzo Gakkai Shi *35*, 349-354.

30. Mesner, L.D., Ray, B., Hsu, Y.H., Manichaikul, A., Lum, E., Bryda, E.C., Rich, S.S., Rosen, C.J., Criqui, M.H., Allison, M., et al. (2014). Bicc1 is a genetic determinant of osteoblastogenesis and bone mineral density. J Clin Invest *124*, 2736-2749. 10.1172/jci73072.
31. Cogswell, C., Price, S.J., Hou, X., Guay-Woodford, L.M., Flaherty, L., and Bryda, E.C. (2003). Positional cloning of jcpk/bpk locus of the mouse. Mamm Genome *14*, 242-249. 10.1007/s00335-002-2241-0.
32. Chittenden, L., Lu, X., Cacheiro, N.L., Cain, K.T., Generoso, W., Bryda, E.C., and Stubbs, L. (2002). A new mouse model for autosomal recessive polycystic kidney disease. Genomics *79*, 499-504. 10.1006/geno.2002.6731.
33. Price, S.J., Chittenden, L.R., Flaherty, L., O'Dell, B., Guay-Woodford, L.M., Stubbs, L., and Bryda, E.C. (2002). Characterization of the region containing the *jcpk* PKD gene on mouse Chromosome 10. Cytogenet Genome Res *98*, 61-66. 10.1159/000068534.
34. Flaherty, L., Bryda, E.C., Collins, D., Rudofsky, U., and Montgomery, J.C. (1995). New Mouse Model for Polycystic Kidney-Disease with Both Recessive and Dominant Gene Effects. Kidney International *47*, 552-558. DOI 10.1038/ki.1995.69.
35. Flaherty, L., Messer, A., Russell, L.B., and Rinchik, E.M. (1992). Chlorambucil-induced mutations in mice recovered in homozygotes. Proc Natl Acad Sci U S A *89*, 2859-2863. 10.1073/pnas.89.7.2859.
36. Schrick, J.J., Vogel, P., Abuin, A., Hampton, B., and Rice, D.S. (2006). ADP-ribosylation factor-like 3 is involved in kidney and photoreceptor development. Am J Pathol *168*, 1288-1298. 10.2353/ajpath.2006.050941.
37. Vogel, P., Gelfman, C.M., Issa, T., Payne, B.J., Hansen, G.M., Read, R.W., Jones, C., Pitcher, M.R., Ding, Z.M., DaCosta, C.M., et al. (2015). Nephronophthisis and retinal degeneration in tmem218-/- mice: a novel mouse model for Senior-Løken syndrome? Vet Pathol *52*, 580-595. 10.1177/0300985814547392.
38. Rachel, R.A., Yamamoto, E.A., Dewanjee, M.K., May-Simera, H.L., Sergeev, Y.V., Hackett, A.N., Pohida, K., Munasinghe, J., Gotoh, N., Wickstead, B., et al. (2015). CEP290 alleles in mice disrupt tissue-specific cilia biogenesis and recapitulate features of syndromic ciliopathies. Hum Mol Genet *24*, 3775-3791. 10.1093/hmg/ddv123.
39. Leightner, A.C., Hommerding, C.J., Peng, Y., Salisbury, J.L., Gainullin, V.G., Czarnecki, P.G., Sussman, C.R., and Harris, P.C. (2013). The Meckel syndrome protein meckelin (TMEM67) is a key regulator of cilia function but is not required for tissue planar polarity. Hum Mol Genet *22*, 2024-2040. 10.1093/hmg/ddt054.
40. Cook, S.A., Collin, G.B., Bronson, R.T., Naggert, J.K., Liu, D.P., Akeson, E.C., and Davisson, M.T. (2009). A mouse model for Meckel syndrome type 3. J Am Soc Nephrol *20*, 753-764. 10.1681/ASN.2008040412.
41. Garcia-Gonzalo, F.R., Corbit, K.C., Sirerol-Piquer, M.S., Ramaswami, G., Otto, E.A., Noriega, T.R., Seol, A.D., Robinson, J.F., Bennett, C.L., Josifova, D.J., et al. (2011). A transition zone complex regulates mammalian ciliogenesis and ciliary membrane composition. Nat Genet *43*, 776-784. 10.1038/ng.891.
42. Bakey, Z., Bihoreau, M.T., Piedagnel, R., Delestre, L., Arnould, C., de Villiers, A., Devuyst, O., Hoffmann, S., Ronco, P., Gauguier, D., and Lelongt, B. (2015). The SAM domain of ANKS6 has different interacting partners and mutations can induce different cystic phenotypes. Kidney Int *88*, 299-310. 10.1038/ki.2015.122.
43. Czarnecki, P.G., Gabriel, G.C., Manning, D.K., Sergeev, M., Lemke, K., Klena, N.T., Liu, X., Chen, Y., Li, Y., San Agustin, J.T., et al. (2015). ANKS6 is the critical activator of NEK8 kinase in embryonic situs determination and organ patterning. Nat Commun *6*, 6023. 10.1038/ncomms7023.

44. Atala, A., Freeman, M.R., Mandell, J., and Beier, D.R. (1993). Juvenile cystic kidneys (jck): a new mouse mutation which causes polycystic kidneys. Kidney Int *43*, 1081-1085. 10.1038/ki.1993.151.
45. Sun, Y., Zhou, J., Stayner, C., Munasinghe, J., Shen, X., Beier, D.R., and Albert, M.S. (2002). Magnetic resonance imaging assessment of a murine model of recessive polycystic kidney disease. Comp Med *52*, 433-438.
46. Tran, P.V., Talbott, G.C., Turbe-Doan, A., Jacobs, D.T., Schonfeld, M.P., Silva, L.M., Chatterjee, A., Prysak, M., Allard, B.A., and Beier, D.R. (2014). Downregulating hedgehog signaling reduces renal cystogenic potential of mouse models. J Am Soc Nephrol *25*, 2201-2212. 10.1681/ASN.2013070735.
47. Phillips, C.L., Miller, K.J., Filson, A.J., Nürnberger, J., Clendenon, J.L., Cook, G.W., Dunn, K.W., Overbeek, P.A., Gattone, V.H., 2nd, and Bacallao, R.L. (2004). Renal cysts of inv/inv mice resemble early infantile nephronophthisis. J Am Soc Nephrol *15*, 1744-1755. 10.1097/01.asn.0000131520.07008.b3.
48. Olbrich, H., Fliegauf, M., Hoefele, J., Kispert, A., Otto, E., Volz, A., Wolf, M.T., Sasmaz, G., Trauer, T., Reinhardt, R., et al. (2003). Mutations in a novel gene, *NPHP3*, cause adolescent nephronophthisis, tapeto-retinal degenerationand hepatic fibrosis. Nature Genet. *34*, 455-459.
49. Takahashi, H., Calvet, J.P., Dittemore-Hoover, D., Yoshida, K., Grantham, J.J., and Gattone, V.H., 2nd (1991). A hereditary model of slowly progressive polycystic kidney disease in the mouse. J Am Soc Nephrol *1*, 980-989. 10.1681/ASN.V17980.
50. Janaswami, P.M., Birkenmeier, E.H., Cook, S.A., Rowe, L.B., Bronson, R.T., and Davisson, M.T. (1997). Identification and genetic mapping of a new polycystic kidney disease on mouse chromosome 8. Genomics *40*, 101-107. 10.1006/geno.1996.4567.
51. Vogler, C., Homan, S., Pung, A., Thorpe, C., Barker, J., Birkenmeier, E.H., and Upadhya, P. (1999). Clinical and pathologic findings in two new allelic murine models of polycystic kidney disease. J Am Soc Nephrol *10*, 2534-2539. 10.1681/ASN.V10122534.
52. Lager, D.J., Qian, Q., Bengal, R.J., Ishibashi, M., and Torres, V.E. (2001). The pck rat: a new model that resembles human autosomal dominant polycystic kidney and liver disease. Kidney Int *59*, 126-136. 10.1046/j.1523-1755.2001.00473.x.
53. Masyuk, T.V., Huang, B.Q., Ward, C.J., Masyuk, A.I., Yuan, D., Splinter, P.L., Punyashthiti, R., Ritman, E.L., Torres, V.E., Harris, P.C., and LaRusso, N.F. (2003). Defects in cholangiocyte fibrocystin expression and ciliary structure in the PCK rat. Gastroenterology *125*, 1303-1310. 10.1016/j.gastro.2003.09.001.
54. Gattone, V.H., 2nd, Tourkow, B.A., Trambaugh, C.M., Yu, A.C., Whelan, S., Phillips, C.L., Harris, P.C., and Peterson, R.G. (2004). Development of multiorgan pathology in the *wpk* rat model of polycystic kidney disease. Anat Rec A Discov Mol Cell Evol Biol *277*, 384-395. 10.1002/ar.a.20022.
55. Nauta, J., Goedbloed, M.A., Van Herck, H., Hesselink, D.A., Visser, P., Willemsen, R., Van Dokkum, R.P.E., Wright, C.J., and Guay-Woodford, L.M. (2000). New rat model that phenotypically resembles autosomal recessive polycystic kidney disease. Journal of the American Society of Nephrology *11*, 2272-2284.
56. Smith, U.M., Consugar, M., Tee, L.J., McKee, B.M., Maina, E.N., Whelan, S., Morgan, N.V., Goranson, E., Gissen, P., Lilliquist, S., et al. (2006). The transmembrane protein meckelin (*MKS3*) is mutated in Meckel-Gruber syndrome and the wpk rat. Nat Genet *38*, 191-196. 10.1038/ng1713.
57. Tammachote, R., Hommerding, C.J., Sinders, R.M., Miller, C.A., Czarnecki, P.G., Leightner, A.C., Salisbury, J.L., Ward, C.J., Torres, V.E., Gattone, V.H., 2nd, and Harris, P.C. (2009). Ciliary and centrosomal defects associated with mutation and depletion of the Meckel syndrome genes *MKS1* and *MKS3*. Hum Mol Genet *18*, 3311-3323. 10.1093/hmg/ddp272.

58. Bihoreau, M.T., Ceccherini, I., Browne, J., Kranzlin, B., Romeo, G., Lathrop, G.M., James, M.R., and Gretz, N. (1997). Location of the first genetic locus, PKDr1, controlling autosomal dominant polycystic kidney disease in Han:SPRD cy/+ rat. Hum Mol Genet *6*, 609-613. 10.1093/hmg/6.4.609.
59. Gretz, N., Kränzlin, B., Pey, R., Schieren, G., Bach, J., Obermüller, N., Ceccherini, I., Klöting, I., Rohmeiss, P., Bachmann, S., and Hafner, M. (1996). Rat models of autosomal dominant polycystic kidney disease. Nephrol Dial Transplant *11 Suppl 6*, 46-51. 10.1093/ndt/11.supp6.46.
60. Kaspareit-Rittinghausen, J., Deerberg, F., Rapp, K.G., and Wcislo, A. (1990). A new rat model for polycystic kidney disease of humans. Transplant Proc *22*, 2582-2583.
61. Kränzlin, B., Schieren, G., and Gretz, N. (1997). Azotemia and extrarenal manifestations in old female Han:SPRD (cy/+) rats. Kidney Int *51*, 1160-1169. 10.1038/ki.1997.159.
62. McCooke, J.K., Appels, R., Barrero, R.A., Ding, A., Ozimek-Kulik, J.E., Bellgard, M.I., Morahan, G., and Phillips, J.K. (2012). A novel mutation causing nephronophthisis in the Lewis polycystic kidney rat localises to a conserved RCC1 domain in Nek8. BMC Genomics *13*, 393. 10.1186/1471-2164-13-393.
63. Ta, M.H., Rao, P., Korgaonkar, M., Foster, S.F., Peduto, A., Harris, D.C., and Rangan, G.K. (2014). Pyrrolidine dithiocarbamate reduces the progression of total kidney volume and cyst enlargement in experimental polycystic kidney disease. Physiol Rep *2*. 10.14814/phy2.12196.
64. Celentano, S., Capolongo, G., and Pollastro, R.M. (2019). [Bardoxolone: a new potential therapeutic agent in the treatment of autosomal dominant polycystic kidney disease?]. G Ital Nefrol *36*.
